# Structural Basis of IL-1 Family Cytokine Signaling

**DOI:** 10.3389/fimmu.2019.01412

**Published:** 2019-06-20

**Authors:** James K. Fields, Sebastian Günther, Eric J. Sundberg

**Affiliations:** ^1^Institute of Human Virology, University of Maryland School of Medicine, Baltimore, MD, United States; ^2^Department of Microbiology & Immunology, University of Maryland School of Medicine, Baltimore, MD, United States; ^3^Program in Molecular Microbiology & Immunology, University of Maryland School of Medicine, Baltimore, MD, United States; ^4^Deutsches Elektronen-Synchrotron DESY, Hamburg, Germany; ^5^Department of Medicine, University of Maryland School of Medicine, Baltimore, MD, United States

**Keywords:** structure, IL-1, IL-33, IL-36, IL-18

## Abstract

Interleukin-1 (IL-1) family cytokines are key signaling molecules in both the innate and adaptive immune systems, mediating inflammation in response to a wide range of stimuli. The basic mechanism of signal initiation is a stepwise process in which an agonist cytokine binds its cognate receptor. Together, this cytokine-receptor complex recruits an often-common secondary receptor. Intracellularly, the Toll/IL-1 Receptor (TIR) domains of the two receptors are brought into close proximity, initiating an NF-κB signal transduction cascade. Due to the potent inflammatory response invoked by IL-1 family cytokines, several physiological mechanisms exist to inhibit IL-1 family signaling, including antagonist cytokines and decoy receptors. The numerous cytokines and receptors in the IL-1 superfamily are further classified into four subfamilies, dependent on their distinct cognate receptors—the IL-1, IL-33, and IL-36 subfamilies share IL-1RAcP as their secondary receptor, while IL-18 subfamily utilizes a distinct secondary receptor. Here, we describe how structural biology has informed our understanding of IL-1 family cytokine signaling, with a particular focus on molecular mechanisms of signaling complex formation and antagonism at the atomic level, as well as how these findings have advanced therapeutics to treat some chronic inflammatory diseases that are the result of dysregulated IL-1 signaling.

## Introduction

During the hunt for the fever-inducing molecule produced by lymphocytes in the second half of the last century, interleukin 1 (IL-1) was discovered (1). Originally given different names, such as leukocytic pyrogen and lymphocyte activating factor, a unifying nomenclature was introduced and it was named interleukin for its capacity to communicate between leukocytes (2). Later, it was discovered that IL-1 exerts its effects on a much broader set of cells, not only leukocytes (3). The purified cytokine had unprecedented activity even at pM levels (1). During biochemical characterization, it was soon realized that these IL-1 purifications contained, in fact, two proteins of similar molecular weight, later named IL-1α and IL-β, that work through the same receptor on cells, inducing similar immunological effect (4). Cloning of the corresponding genes and subsequent recombinant expression of the proteins paved the way for detailed molecular studies.

With the improvement of genome analysis during the 1990s, the number of genes that could be identified as IL-1-like cytokines grew substantially. Now, 11 cytokines and 10 receptors are considered members of this cytokine family (Tables 1, 2) (5). For the majority of cases, the genomic identification of IL-1 family members preceded the discovery of their immunological function. In fact, there are still cytokines whose modes of action are not entirely clear (e.g., IL-37, IL-38) and receptors whose ligands and/or function are not yet fully described (e.g., SIGIRR, IL-1RAPL1/2).

**Table 1:** List of IL-1 family cytokines with their respective nomenclatures, Uniprot IDs, alternative names, domains, and structures by PDB code.

**Cytokines**	**Uniprot**	**Alternative name**	**Main Domain**	**Structure**	**Structure**	**Structure**	**Structure**	**Structure**
IL1-F1	P01583	IL-1α	113-271	2ILA (Cα only)	2KKI (NMR)			
IL1-F2	P01584	IL-1β	117-269	2I1B	6I1B(NMR)	1ITB (with IL-1RI)	4DEP (with IL-1RI/IL-1RAcP)	3O4O (with IL-1RII/ IL1-RAcP)
IL1-F3	P18510	Il-1Ra, anakinra	26-177	1ILR	1IRP (NMR)			
IL1-F4	Q14116	IL-18	37-193	2VXT (with antibody)	1J0S (NMR)	3F62 (with IL-18BP)	3WO4 (IL-18R/IL-18RAcP/IL-18)	3WO3 (IL-18R/IL-18RAcP/IL-18)
IL-1F5	Q9UBH0	IL-36Ra	1-155	1MD6 (murine)	4P0J (chimera)	4P0K (chimera)	4P0L (chimera)	
IL-1F6	Q9UHA7	IL-36α	1-158					
IL-1F7	Q9NZH6	IL-37	46-218	5HN1				
IL-1F8	Q9NZH7	IL-36β, IL1-H2	1-157					
IL-1F9	Q9NZH8	IL-36γ	1-169	4IZE				
IL1-F10	Q8WWZ1	IL-38	1-152	5BOW				
IL-1F11	O95760	IL-33, NF-HEV	112-270	2KLL (NMR)	4KC3 (ST2/IL-33)			

**Table 2:** List of IL-1 family cytokine receptors with their respective nomenclatures, Uniprot IDs, alternative names, domains, and structures by PDB code.

**Receptor**	**Uniprot**	**Alternative name**	**Structure**	**Structure**	**Structure**
**PRIMARY RECEPTORS**					
IL-1R1	P14778	IL-1RI, CD121a	1ITB (with IL-1β)	1G0Y (with antagonistic peptide)	4DEP (IL-1β/IL-1RI/IL-1RAcP)
IL-1R2	P27930	IL-1RII, CD121b	3O4O (IL-1β/IL-1RII/IL-1RAcP)		
IL-1R4	Q01638	IL-33R, ST2, IL1-RL1, DER4, T1, IL-1R4	4KC3 (ST2/IL-33)	5VI4 (ST2/IL-1RAcP/IL-33)	
IL-1R5	Q13478	IL-18Rα, IL-1Rrp, IL-1Rrp1	3WO4 (IL-18/IL-18Rα/IL-18Rβ)	3WO3 (IL-18/IL-18Rα)	4R6U (IL-18/IL-18Rα)
IL-1R6	Q9HB29	IL-36R, IL-1RL2, IL-1Rrp2,			
**CO-RECEPTORS**					
IL-1R3	Q9NPH3	IL-1RAcP	4DEP (IL-1β/IL-1RI/IL-1RAcP)	3O4O (IL-1β/IL-1RII/IL-1RAcP)	
IL-1Rb		IL-1RAP			
IL-1R7	O95256	IL-18Rβ, AcPL	3WO4 (IL-18/IL-18Rα/IL-18Rβ)	3WO3 (IL-18/IL-18Rα)	4R6U (IL-18/IL-18Rα)
IL-1R8	Q6IA17	SIGGIR, TIR8			
IL-1R9	Q9NZN1	IL-1RAPL1 OPHN4, TIGIRR-2	5WY8 (IL-1RAPL1/PTPRdelta 1T3G (TIR)	4M92 (peptide 207-222 in complex with N33/Tusc3)	
IL-1R10	Q9NP60	IL-1RAPL2, TIGIRR-1			

Structural biology has been instrumental in answering some of the central questions concerning IL-1 family cytokine signaling. For example, despite the similarity in function, sequence identity of the mature cytokines IL-1α and IL-1β is only 25%. With the solution of the X-ray crystal structures of both cytokines, it became evident that both mature cytokines share an overall fold, explaining the ability to engage the same receptor (4, 6). Later, the structure of the naturally occurring antagonist cytokine IL-1 receptor antagonist (IL-1Ra) was found to exhibit the same fold as both agonist cytokines, IL-1α and IL-1β. Comparison of the IL-1 receptor (IL-1RI) bound to IL-1β and IL-1Ra, combined with earlier mutagenesis work, revealed how IL-1Ra can compete with IL-1β for binding its primary receptor yet prevent engagement of the co-receptor IL-1 receptor accessory protein (IL-1RAcP) (7, 8) and, thus, inhibit IL-1 signaling. Another long-standing question was how the binary receptor-cytokine pair can engage its co-receptor IL-1RAcP, the final step of signal initiation. It was not until the structure of the ternary complex of IL-1β with a decoy receptor, IL1RII, and its co-receptor, IL-1RAcP, was published that this was finally clarified (9).

Guided by structural studies, we now have a detailed picture of the general mechanisms of signal activation and inhibition in the IL-1 cytokine family, of which we describe key features in more detail in the following sections.

## Signaling

All members of the IL-1 family are extremely potent modulators of inflammation. Hence, their activities are regulated on several levels, including gene transcription, expression as inactive proforms, secretion and binding at the receptor level. Almost all cytokines of the IL-1 family are expressed as proforms with N-terminal domains of varying length from more than 100 amino acids (IL-33) to just a single amino acid (IL-36Ra). Proteases remove the N-terminal amino acids, creating mature, signaling-competent cytokines (10, 11). Once active cytokines are secreted, they can bind to their cognate cell surface receptors and initiate signaling. Within the IL-1 family, the mechanism of signal initiation is highly conserved.

**Figure 1:**
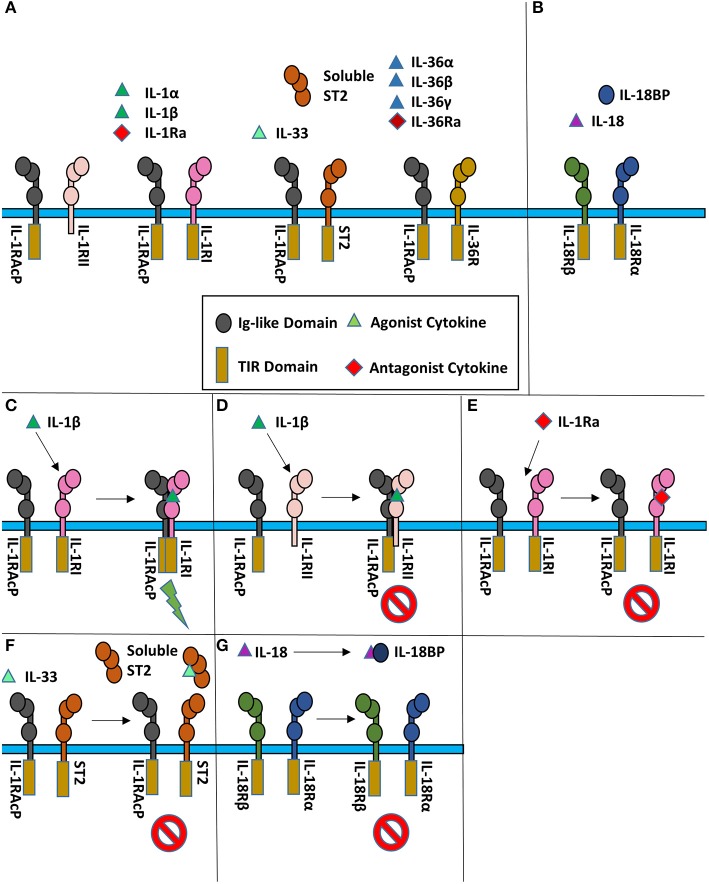
Cartoon representation of IL-1 super family. **(A)** Cytokines of the IL-1 subfamily are above their respective receptor binding partners. **(B)** IL-18 has a different co-receptor, IL-18Rβ, and is a part of the IL-18 subfamily. **(C)** Upon binding of IL-1β to IL-1RI, IL-1RAcP is recruited to initiate signaling. **(D)** When IL-1β binds IL-1RII, no signaling occurs as IL-1RII lacks a cytoplasmic TIR domain. **(E)** When the IL-1R receptor antagonist (IL-1Ra) binds IL-1RI, the IL-1RAcP is not recruited, leading to no signaling. **(F)** IL-33 signaling can be inhibited by sequestration of the cytokine by the soluble ST2 receptor. **(G)** IL-18 can be sequester by the IL-18 binding protein (IL-18BP) to inhibit signaling.

Typical agonist signaling is initiated by a cytokine, such as IL-1β, binding its cognate receptor IL-1RI with nM affinity (Figure 1). Upon binding, a shared co-receptor, IL-1RAcP, is recruited by binding to the composite surface of the cytokine and primary receptor complex, resulting in the creation of a ternary complex; the binding affinity of IL-1RAcP is approximately 100-fold weaker than that of the IL-1β/IL-1RI complex. Through a single transmembrane helix spanning the plasma membrane, the ectodomains of these receptors are attached to Toll/interleukin-1 receptor (TIR) domains that reside in the cytoplasm. As the trimeric complex containing the cytokine, primary receptor, and accessory protein is formed, the cytoplasmic TIR domains of the two receptors are brought together to elicit downstream signaling via Myd88-dependent signaling pathways.

At the receptor level, signaling can be regulated by antagonistic cytokines. These bind to the primary receptor yet do not allow the accessory receptor to form the trimeric complex, thus prohibiting IL-1 signaling (Figure 1E). This can also be achieved by decoy receptors (Figures 1D,F). These receptors bind the cytokine but lack the intracellular TIR domain necessary for signaling, thereby neutralizing agonist cytokines.

One hallmark of IL-1 signaling is the redundancy of cytokines capable of binding the same cognate receptor (12). For instance, the primary receptor IL-1RI binds IL-1α, IL-1β, and IL-1Ra and the inhibitory receptor IL-1RII binds the same three cytokines, albeit with different affinities (13). The four IL-36 cytokines (the agonists IL-36α, IL-36β, and IL-36γ, and the antagonist IL-36Ra) share IL-36R as their single primary receptor. The most promiscuous receptor is IL-1RAcP, the co-receptor for three primary receptors, one decoy receptor and six agonist cytokines, binding eight different cytokine/receptor pairs altogether.

## Cytokines

**Figure 2:**
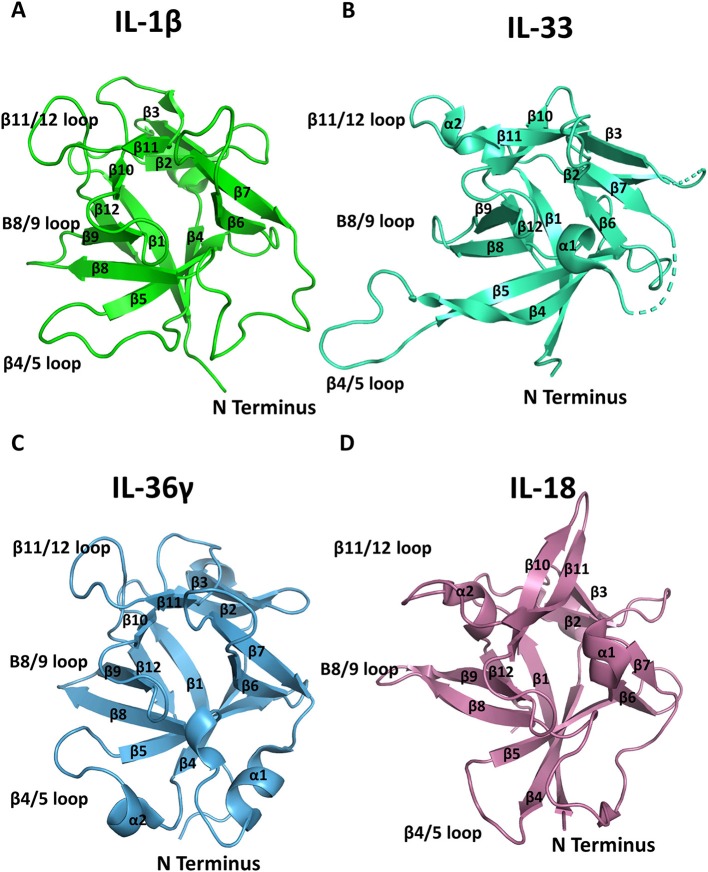
Representation of cytokine structures. **(A)** The structure of IL-1β with its β-sheets and key loops labeled. **(B)** The structure of IL-33 with its β-sheets and key loops labeled. **(C)** The structure of IL-36γ with its β-sheets and key loops labeled. **(D)** The structure of IL-18 with its β-sheets and key loops labeled.

The high-resolution structures of IL-1α, IL-1β, IL-1Ra, IL-33, and IL-36γ, IL−18, IL-37, and IL-38 cytokines have all been determined by either X-ray crystallography or solution state NMR. These cytokines all possess a conserved β-trefoil conformation and a central hydrophobic core composed of 12 β-sheets, six of which (β1, β4, β5, β8, β9, and β12) form an anti-parallel β-barrel (Figure 2) (14). The β-trefoil consists of six β-hairpins and, using the structure of IL-1β by way of example, the naming of the β-sheets starts consecutively from the N-terminus for all IL-1 family cytokines (Figure 2A). While this structural motif is conserved among the cytokines, their sequence identity is low, even for members that bind the same primary receptor. Due to the inherent affinities for their primary receptors, these cytokines function at picomolar levels in order to elicit their downstream effects.

### IL-1

IL-1 is an extremely potent inflammatory cytokine that is involved in myriad immunological responses, spanning both innate and adaptive immunity (15). Of the cytokines that bind the primary receptor IL-1RI, there are two similar yet distinct molecules, IL-1α and IL-1β, which are encoded by different genes. The IL-1α precursor gene is expressed constitutively in cells, including kidney, liver, lung, endothelial cells, astrocytes, and the epithelium of the gastrointestinal track (3). Unlike IL-1β, IL-1α is already active in its primary precursor form and acts as an alarmin by eliciting a signaling cascade through IL-1RI. The crystal structure of IL-1α has been determined at a resolution of 2.7 Å. (4). Similar to other cytokines within the IL-1 family, IL-1α is composed of 12 β-strands in a β-trefoil architecture.

Unlike IL-1α, IL-1β is expressed in a more limited number of cell types and must be processed from its precursor form to become an active agonist in IL-1 signaling. IL-1β is transcribed by monocytes, macrophages, and dendritic cells following Toll-like receptor (TLR) activation by pathogen-associated molecular patterns (PAMPs) or cytokine signaling. IL-1β is also transcribed in the presence of itself in a form of auto-inflammatory induction (15, 16). The inactive IL-1β precursor needs to be processed by caspase-1 cleavage, which in turn requires activation by danger-associated molecular patterns (DAMPs).

While active IL-1β and IL-1α have a sequence identity of only 25%, their overall structures are highly similar with an overall root mean square deviation (RMSD) of 1.54 Å over all Cα positions (17). The β4/5 and β11/12 (Figure 2A) loops of the cytokine are instrumental for their function and differ between agonist and antagonist cytokines, as discussed below.

### IL-33

IL-33, the latest addition to the IL-1 superfamily, was discovered over a decade ago and is now clearly defined as a key component of innate and adaptive immune responses (18, 19). The IL-33 receptor, ST2, had been discovered previously and was considered an orphan receptor in the absence of any known ligand. ST2 was first used as a marker to differentiate T helper 2 (Th2) from T helper 1 (Th1) cells as it was selectively expressed on the former. Later, the ST2/IL-1RAcP signaling complex was shown to exist on group 2 innate lymphoid (ILC2) cells, helper and regulatory T cells, mast cells, basophils, eosinophils, NKT cells, and NK cells (20). As such, this cytokine is instrumental in immune defense against parasites and viruses (21–23).

Similar to IL-1α, IL-33 is biologically active in its nuclear form and is expressed constitutively in tissues, although subsequent cleavage by proteases can increase its potency (24, 25). Conversely to IL-1β, IL-33 is inactivated by caspase-1 cleavage during apoptosis (26). It acts as an alarmin critical to innate and adaptive immune defenses. IL-33 also plays an important role in allergic inflammation. Upon allergen induced activation, IL-33 protein levels increase beyond its basal levels (27–29). Unlike IL-1, there is no known antagonist cytokine to downregulate this activation. Instead, ST2 also exists in a soluble form (soluble ST2, sST2) that contains only the ectodomain of the receptor, composed of three immunoglobulin (Ig) fold domains, with no transmembrane helix (Figure 1F). As this decoy receptor is released, excess IL-33 may be sequestered to limit IL-33 driven inflammation.

The IL-33 fold was first predicted by a computational screen based on structural alignments of IL-1 family cytokines and fibroblast growth factor (FGF) β-trefoil cytokines; its three-dimensional structure was then determined by NMR (30). Similar to both IL-1α and IL-1β, IL-33 is a 12-stranded β-barrel surrounding a hydrophobic core in a β-trefoil configuration. There are two alpha helices (α1 and α2) that precede β-strands β8 and β12 (Figure 2B). The β 4/5 loop of IL-33 is substantially longer than those of other IL-1 family cytokines with an additional 10 amino acids (30).

### IL-36

The IL-36 receptor (IL-36R) is the most promiscuous primary receptor in the IL-1 family; it binds three agonist cytokines, IL-36α, IL-36β, and IL-36γ, as well as a single antagonist cytokine, or receptor antagonist, IL-36Ra. IL-36R, and the IL-36 cytokines were discovered separately through genome screening (31, 32). Both remained orphaned until their functional dependence was shown (33). While IL-36α, IL-36β, IL-36γ, and IL-36Ra all lack an N-terminal secretion signal, they are secreted by an unidentified mechanism as they function, putatively, extracellularly (34).

While relatively new to the IL-1 family, the broader immunological role of the IL-36 cytokines began to be elucidated after the discovery that their N-terminus must be processed precisely for higher affinity binding to its cognate receptor (35). CD4+ T cells, upon activation, may be stimulated by IL-36 agonist cytokines to induce IL-2 production, proliferate, and be subsequently polarized for a Th1 response (36). In addition to T cells, IL-36 cytokines are involved in the regulation of dendritic cells (37). IL-36γ is the only agonist cytokine of the IL-36 subfamily for which a high-resolution structure has been determined (Figure 2C) (38).

### IL-18

IL-18 is best known for its capacity to induce IFN-γ and is expressed by macrophages, epithelial cells, such as keratinocytes, and dendritic cells (34). Reminiscent of IL-1β, IL-18 must be processed from its 23 kDa proform by caspase-1 into its 18 kDa active form (39). The architecture of IL-18 is grossly similar to those of the other IL-1 family member cytokines (Figure 2D).

## Binary Complexes

**Figure 3:**
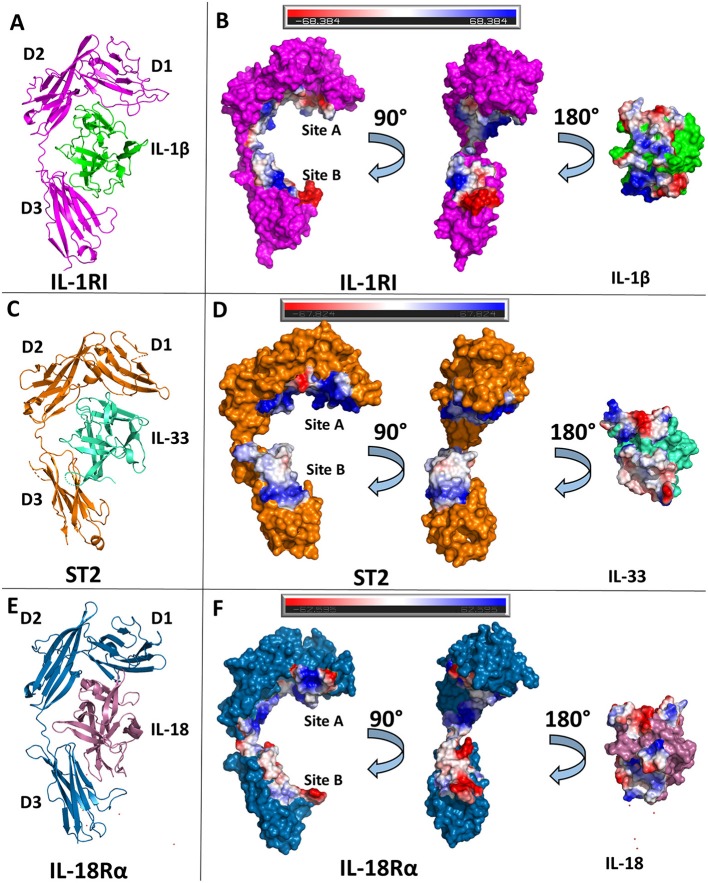
Structures of binary cytokine/receptor complexes. **(A)** Cartoon of IL-1β/IL-1RI binary complex (pdb: 1itb). **(B)** Surface of both IL-1β and IL-1RI with respective interfaces showing electrostatic potential. **(C)** Cartoon of IL-33/ST2 binary complex (pdb: 4kc3). **(D)** Surface of both IL-33 and ST2 with respective interfaces showing electrostatic potential. **(E)** Cartoon of IL-18/IL-18Rα binary complex (pdb: 3wo3). **(F)** Surface of both IL-18 and IL-18Rα with respective interfaces showing electrostatic potential.

The formation of the binary complex is a key step in the initiation of a functioning signaling complex. Through structural studies, much has been elucidated concerning these interactions. The primary receptors share a similar overall molecular architecture and bind their respective cytokines in conserved binding sites (Figure 3). Due to these similarities, we use the structure of IL-1β bound to IL-1RI here to highlight the commonalities between all three binary complex structures.

### IL-1β/IL-1RI

The ectodomain of the primary receptor IL-1RI contains three Ig-like domains (D1, D2, and D3) that form two distinct binding sites, A and B, that together drive its interactions with IL-1 cytokines (Figure 3B). The primary receptor adopts an architecture that resembles a grasping hand in how it binds the cytokine. D1 and D2 are tightly packed against each other and, together, their contiguous molecular surface constitutes binding site A. Between D1/D2 and D3, there is a 6 amino acid linker lacking secondary structure. D3 is a single Ig domain and forms binding site B (8). IL-1β, in total, has a buried surface area within the interface of IL-1RI of 1932 Å^2^ over 47 residues. When divided between respective binding sites, IL-1β has a buried surface area of ~1,000 Å^2^ over 25 amino acids at site A (8). In site B, formed by the D3 domain of the receptor, there is a nearly equivalent sized interface to the D1/D2-IL-1β interface over 21 amino acids (8). Five of the six β-sheets from the Ig fold of D3 are involved in this interface. Additionally, there is a hydrogen bond between IL-1β with the linker between D1/2 and D3, falling outside the canonical sites A and B.

### IL-33/ST2

IL-33 binds its primary receptor, ST2, with an affinity of 450 pM (30). While a crystal structure of the binary complex IL-33/ST2 was determined in 2013 (40), a previous NMR structure of IL-33 alone had been published in 2009 (30). IL-33 exhibits very little conformational change upon binding ST2; there is an RMSD of 1.2 Å over all Cα atoms between ST2-bound and unbound IL-33. As with IL-1RI, ST2 is composed of three Ig domains, all of which interact with IL-33, that can be further divided into site A and B, analogous to IL-1RI/IL-1β (40).

While the overall D1/D2 architecture is conserved between the known structures of IL-1β/IL-1RI and IL-33/ST2, the orientation of the D3 domain relative to D1/2 is not, resulting in an RMSD of 4.51 Å between the two receptors. Together, IL-33 and ST2 share a buried surface area of roughly 1,700 Å^2^. When divided into the respective sites, site A of ST2 has a buried surface area of 940 Å^2^ contributed by 30 residues. The vast majority of this interface has a positive electrostatic potential (Figure 3D). On the D3 of ST2, binding site B, there is a buried surface area of 818 Å^2^ over 22 residues. Binding site B, in contrast to site A, has a lower electrostatic potential and relies heavily on salt bridges between ST2 and IL-33.

### IL-18/IL-18Rα

Shortly after the publication of the IL-33/ST2 crystal structure, the binary complex of IL-18 with its primary receptor IL-18Rα was published (41). IL-18Rα, like the other primary receptors within the IL-1 family, is composed of three Ig-like domains that can be grouped into two respective parts, D1/D2 and D3. In comparison to the IL-1β/IL-1RI and IL-33/ST2 binary structures, the IL-18/IL-18Rα structure has a similar overall architecture of its domains. As with the other cytokine/primary receptor pairs, IL-18 binds to IL-18Rα at two distinct sites, encompassing all three Ig domains of the primary receptors (Figure 3F). In total, IL-18 has a buried surface area with IL-18Rα of 1,650 Å^2^ over 49 residues. From the perspective of IL-18Rα, binding site A has a buried surface area of 890 Å^2^ over 30 AA. This is composed mainly by residues on the β1/2 loop, β2 and β3 strands, and β10/11 loops of IL-18 (Figure 2D). As shown by the crystal structures, hydrophilic interactions dominate this interaction. When considering binding site B on IL-18Rα, there are six residues from IL-18 that make up the composition of this interface. On IL-18Rα, site B is composed of 22 amino acids. This, in total, results in a buried surface area of roughly 600 Å^2^ (41).

## Ternary Complexes

The available ternary complex structures of the IL-1 family members all share common structural motifs. This is not wholly surprising. For one, a feature of the IL-1 family is the redundancy of binding partners. Highly variable cytokine sequences result in a common secondary structure. In turn, these cytokines can bind to the same primary receptors at nM affinity (e.g., similar affinities of IL-1α and IL-1β for IL-1RI). The primary and secondary structures are both composed of three Ig-like domains. The binding of the cytokine to the primary receptor creates a composite surface for the recruitment of the secondary receptor. This allows cytoplasmic TIRs to aggregate for a MyD88 signaling cascade. Even for cytokines that share a common secondary-receptor, these protein complexes are able to interact differently with the secondary receptor at the same key areas. This allows the same co-receptor, IL-1RAcP, to be a key mediator of vastly different immunologic outcomes.

### IL-1 Ternary Complexes

The first high-resolution ternary structure in the IL-1 family determined was the inhibitory complex of IL-1β with the decoy receptor IL-1RII and IL-1RAcP (9). Shortly thereafter, the signaling-competent ternary complex IL-1β/IL-1RI/IL-1RAcP was determined (42). The two ternary complexes display high structural similarity to one another with an RMSD of 1.8 Å between their Cα atoms. These structures have greatly informed our understanding of IL-1 family signaling mechanisms by revealing the interactions necessary for the recruitment of the accessory protein.

The overall architecture of the binary complex IL-1β/IL-1RI remains predominately unchanged when the secondary receptor is recruited to form the trimeric complex IL-1β/IL-1RI/IL-1RAcP, with an RMSD of 1.4 Å (42). The binding of the cytokine to its cognate receptor allows for a composite surface between IL-1β/Il-1RI to recruit the accessory protein with sub-μM affinity, as demonstrated by surface plasmon resonance (SPR) (9). No IL-1 cytokine has appreciable affinity for IL-1RAcP on its own in the absence of its cognate receptor.

**Figure 4:**
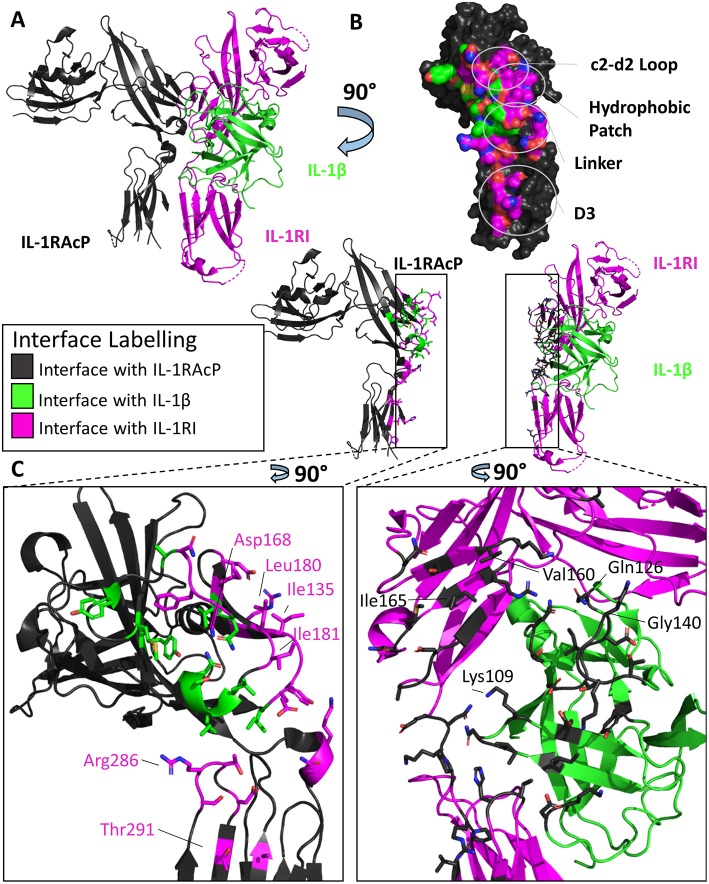
Structure of the IL-1β/IL-1RI/IL-1RAcP ternary complex. **(A)** Cartoon of IL-1β/IL-1RI/IL-1RAcP ternary complex (pdb: 4dep). **(B)** Surface of IL-1RAcP with interface to binary complex labeled and colored according to its binding partner. **(C)** Cartoon of IL-1RAcP and IL-1β/IL-1RI with residues involved in interface shown as sticks colored according to its binding partner.

Previous to the structural determination of these trimeric complexes, the precise orientation of the accessory protein to its respective binary complex was unknown, although attempts to discern its interaction with the binary complex was modeled (43). Similar to its counterpart IL-1RI, the accessory protein is composed of three Ig-like domains whose D1/2 domains are juxtaposed to each other. As seen in Figure 4B, however, the accessory protein binds with its backside to IL-1β/IL-1RI, making extensive contact with IL-1RI in the D2 domain and, to a lesser extent, to the D3 domain. D1 of the accessory protein is located far from the interface and makes no contacts with the binary complex (Figure 4A). The interface corresponding to site A and B in the primary receptors is not involved in binding the binary cytokine/receptor complex. As had been previously described in mutagenesis studies of IL-1β and IL-1Ra, the β4/5 and the β11/12 loops of these cytokines were crucial for their opposing agonist/antagonist functions (44, 45). In subsequent SPR studies, IL-1Ra with loop swaps for IL-1β β4/5 and β11/12 rescued binding of the IL-1Ra/IL-1RI complex to the accessory protein, albeit with a lower affinity (i.e., μM) than the inherent sub-μM affinity the IL-1RAcP has for the IL-1β/IL-1RI binary complex (9), providing a molecular mechanism by which agonist and antagonist cytokines of the IL-1 family function to either recruit IL-1RAcP or not, respectively.

### IL-33 Ternary Complex

**Figure 5:**
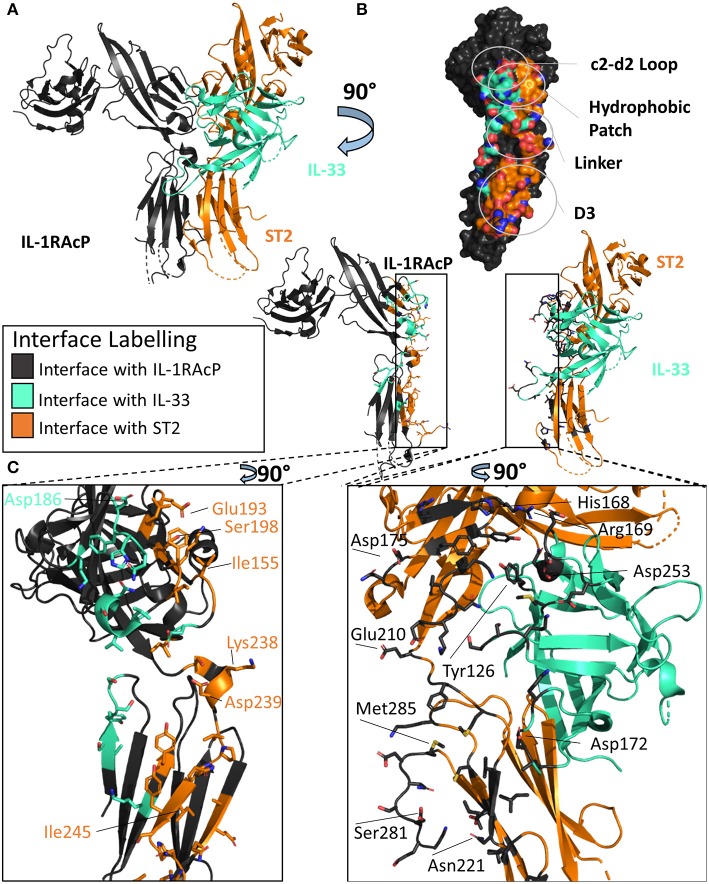
Structure of the IL-33/ST2/IL-1RAcP ternary complex. **(A)** Cartoon of IL-33/ST2/IL-1RAcP ternary complex (pdb: 5vi4). **(B)** Surface of IL-1RAcP with interface to binary complex labeled and colored according to its binding partner. **(C)** Cartoon of IL-1RAcP and IL-33/ST2 with residues involved in interface shown as sticks colored according to its binding partner.

As both the IL-1β/IL-1RI and IL-33/ST2 binary structures described previously had high structural homology when aligned, it was originally thought that these complexes would recruit their shared accessory protein, IL-1RAcP, in a similar fashion (40). When the X-ray crystal structure of the IL-33/ST2/IL-1RAcP was finally determined (Figure 5A), however, the diverse ways in which IL-1 family cytokines could recruit IL-1RAcP were finally appreciated (46).

There are many similarities between the two signaling-competent ternary complexes of IL-1β/IL-1RI/IL-1RAcP and IL-33/ST2/IL-1RAcP. Both ternary complexes contain the same overall structure, with an RMSD of 3.2 Å. Additionally, both binary complexes engage IL-1RAcP on the backside of IL-1RAcP, interacting with the D2 and D3 of the co-receptor. As with IL-1β, the β4/5 and β11/12 of IL-33 loops are at the interface with IL-1RAcP, with a buried surface area of 201 Å^2^ while having a 54 Å^2^ buried surface area to ST2 (Figure 5B).

These two binary complexes engage key regions of the IL-1RAcP differently, however. On IL-1RAcP, there exist four distinct areas that interact with both the IL-1β/IL-1RI and IL-33/ST2 binary complexes: the c2-d2 loop region, the hydrophobic patch, the linker region and the D3 region. The c2-d2 region is a loop connecting strands c and d in the D2 domain of the IL-1RAcP (Figures 4B, 5B). These strands exhibit conformational plasticity, a trait that allows them to interact with both binary complexes in distinct ways to accommodate their inherent differences. For both, however, there exists a network of hydrogen bonds that interacts with the respective binary complexes. The hydrophobic patch is a region on IL-1RAcP that makes hydrophobic interactions with both binary complexes. An important residue within the IL-1RAcP is Ile155, which engages both the cytokine and primary receptor in the case of IL-1β/Il-1RI through shared hydrophobicity and engages ST2 through van der Waals contacts to Asp175 of ST2. The linker region encompasses residues that reside between D2 and D3 of the IL-1RAcP and engage both binary complexes through different composite surfaces. Lastly, the D3 region of the IL-1RAcP engages both binary complexes differently. The D3 of ST2 is rotated by 60 degrees around the long axis of the domain, thereby presenting a larger surface for the D3 of IL-1RAcP than for the IL-1β/IL-1R1 complex (Figures 4C, 5C).

There exist subtle differences in the overall domain positioning upon ternary complex formation as well. For IL-1β/IL-1RI, the D1/2 domain is rotated 3.7 degrees away from the interface with IL-1RAcP while the D3 domain rotated 6.6 degrees toward the interface, resulting in a 3 Å displacement of residues when compared to the binary complex. For IL-33/ST2, the D1/2 domain in contrast rotates toward the IL-1RAcP interface, while the D3 domain similarly rotated by 9 degrees toward the IL-1RAcP D3 domain (46).

To further elucidate key differences between the IL-1RI and ST2 ternary complexes, hydrogen-deuterium exchange coupled to mass spectrometry (HDX-MS) was conducted to assess intrinsic protein flexibility in the ternary complex (46). For ST2, a major peptide fragment that differed in flexibility upon co-receptor binding was peptide 166-172_ST2_ that lay directly on a hydrophobic patch with the IL-1RAcP. Similarly, in the IL-1β/IL-1RI/IL-1RAcP ternary complex, fragment 129-142_IL−1RI_, part of a hydrophobic patch, exhibited reduced flexibility, indicative of binding of IL-1RAcP (46).

A key difference in the HDX-MS data that highlighted the intrinsic differences between recruitment of IL-1RAcP by the binary complex involved loops β4/5 and β11/12 of the cytokine. The β4/5 and β11/12 loops of IL-1β make key interactions with the IL-1RAcP. IL-1Ra has differences within these loops that do not allow the recruitment of the Il-1RAcP for a functioning ternary complex. This importance does not translate to the IL-33 ternary structure, however. In the β11/12 regions, this loop was highly shielded from exchange in the IL-1β ternary complex; the overall exchange was half (46). As both the β4/5 and β11/12 loops of IL-33 showed a large amount of exchange over a long time scale, these areas are clearly not tightly engaged in the ternary structure.

Through extensive alanine scanning mutagenesis, hotspots of IL-1RAcP recruitment for both IL-1β/IL-1RI and IL-33/ST2 were identified (46). It was from these studies that inherent differences were highlighted between these respective binary complexes. IL-1RI displayed a narrow distribution of binding energy, localized to key residues; ST2 displayed a broader distribution of binding over more residues.

**Figure 6:**
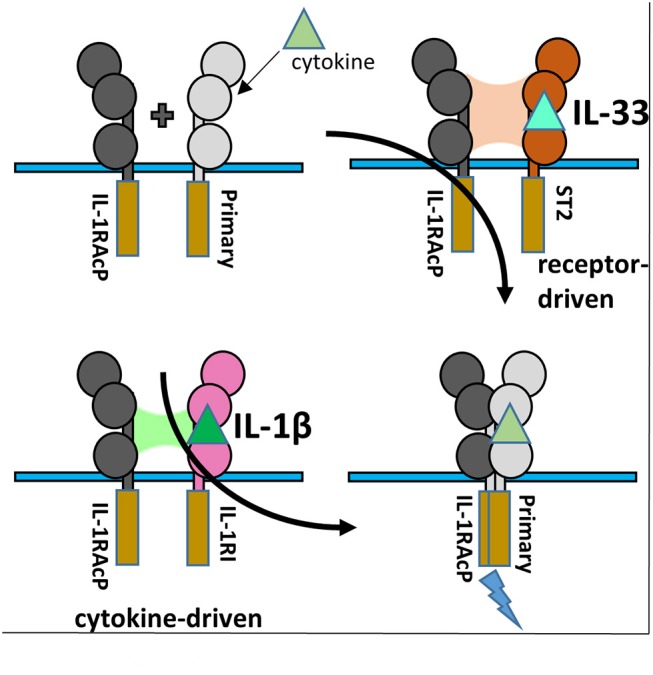
IL-1RAcP Recruitment by IL-1β/IL-1RI and IL-33/ST2 binary complexes. **(A)** Cartoon of the differences inherent in IL-1RAcP recruitment by IL-1β/IL-1RI and IL-33/ST2 binary complexes.

Although interacting with the same key regions of IL-1RAcP, it is clear that there exist marked differences in the recruitment of the IL-1RAcP between the IL-1β and IL-33 ternary complexes. While for IL-lβ/IL-1R1/IL-1RAcP, the cytokine is the main driving force in the interaction with the co-receptor, for IL-33/ST2/IL-1RAcP it is the primary receptor (Figure 6). In this case, the cytokine seemingly only arrests the primary receptor ST2 in a conformation that enables its interaction with IL-1RAcP (46).

### IL-18 Ternary Complex

**Figure 7:**
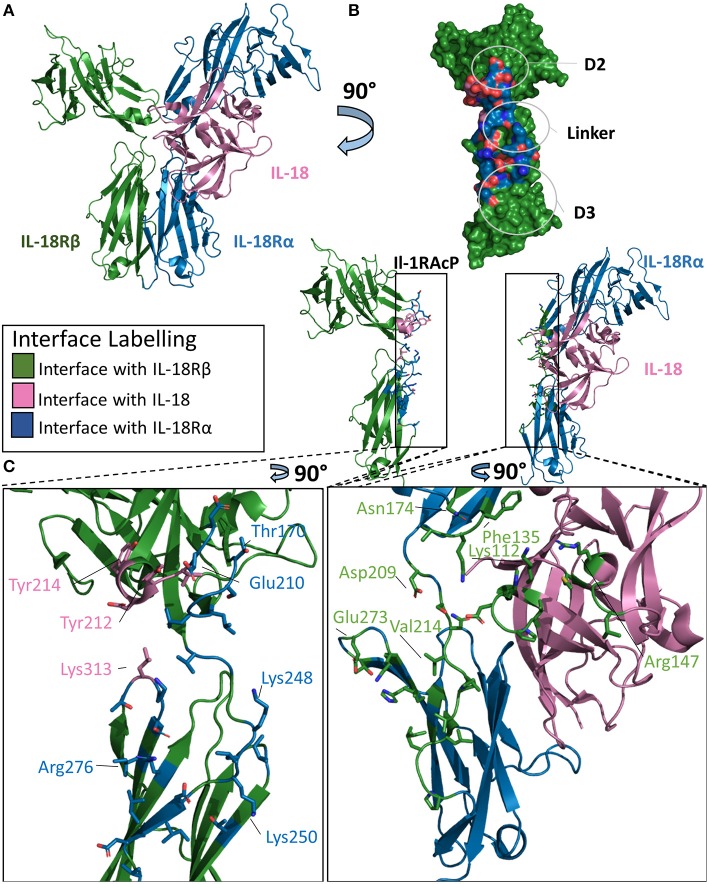
Structure of the IL-18/IL-18Rα/IL-18Rβ ternary complex. **(A)** Cartoon of IL-18/IL-18Rα/IL-18Rβ ternary complex. **(B)** Surface of IL-18Rβ with interface to binary complex labeled and colored according to its binding partner. **(C)** Cartoon of IL-1RAcP and IL-18/IL-18Rα with residues involved in interface shown as sticks colored according to its binding partner.

The IL-18 ternary complex provided new insight into the function of IL-1 family members that do not share the IL-1RAcP (47). The IL-18 ternary complex forms when the binary complex IL-18/IL-18Rα is recognized by the IL-18Rβ secondary receptor (Figure 7A). As with other ternary complexes, the IL-18Rβ will not bind IL-18Rα prior to formation of the binary IL-18/IL-18Rα complex (48).

The overall architecture of the IL-18/IL-18Rα/IL-18Rβ structure is highly similar to that of the ternary complexes involving IL-1β. The cytokine binds the primary receptor and presents a shared surface for the secondary receptor. The secondary receptor is composed of three Ig-like domains, interacting with the binary complex through the D2 and D3 domains. IL-18 binds IL-18Rα with a low nM affinity (20–40 nm), while the recruitment of the IL-18Rβ forms the larger ternary complex at high nM affinity (49).

This high affinity complex has multiple interactions with both the cytokine and the IL-18Rα chain. For the cytokine, there are 12 residues in the interface with the IL-18Rβ chain, composing a buried surface area of 453 Å^2^. This constitutes 5.3% of the solvent accessible area of the cytokine. The entirety of this interface is composed of hydrogen bonds over those 15 residues on IL-18. For the IL-18Rβ, this interface is roughly 380 Å^2^ in size and is composed of 11 residues. This is reminiscent of IL-1β's role in the formation of the IL-1 ternary complex with Il-1RAcP.

IL-18Rα grasps IL-18 in a similar fashion to the IL-1β/IL-1RI binary complex and has a similar orientation of the accessory protein for the respective IL-18Rβ. On ternary complex formation, the binary complex does not change significantly, with an RMSD of 0.7 Å. Additionally, the co-receptor IL-18Rβ adopts an orientation reminiscent of IL-1RAcP in the IL-1β ternary complex. The RMSD between these ternary structures is 4.6 Å.

There are, however, major differences in the positions of the D2 and D3 domains to the IL-18Rβ in comparison the IL-1β ternary complex. IL-18Rα D2 supplies two loops at the interface of IL-18Rβ D2, namely B2 and E3, that interact together. In addition to electrostatic interactions seen in IL-1β/IL-1RAcP, the IL-18/18Rβ interface has aromatic interactions that contribute to the affinity of the ternary complex. The β4/5 loop of IL-18, however, does not interact with IL-18Rβ as it does within the IL-1β/IL-1RAcP interaction (Figure 7B).

Recognition of the binary complex IL-18/IL-18Rα by IL-18Rβ is mediated by numerous interactions (Figure 7C). In the crystal structure, Tyr212 of IL-18Rβ makes aromatic interactions, most likely pi-stacking from a 3.4 Å distance, with IL-18 in a core concave area of the D2 interactions shared between IL-18 and IL-18Rα (47). Overall, there are 14 residues from IL-18Rα that interact with IL-18Rβ, resulting in a buried surface area of 511 Å^2^. Conversely, 25 residues of IL-18Rβ recognize IL-18Rα, constituting an interface surface area of 802 Å^2^.

Analysis of the interface size of the three ternary complex structures revealed that the size of the interface formed by the cytokine together with the primary receptor D1/2 domains and secondary receptor is constant (~2,000 Å^2^), while the overall interface size varies from about 2,500 Å^2^ for IL-1β/IL-1RII/IL-1RAcP to over 3,700 Å^2^ for IL-33/ST2/IL-1RAcP due to the different contribution by the primary receptor D3 domain (46). In contrast, the interface in the IL-18 complex with its unique coreceptor IL-18Rβ has the overall smallest interface between the secondary receptor and binary cytokine/primary receptor complex (2,400 Å^2^). Although the supramolecular structures are inherently similar, their exist differences between the three known ternary structures.

## Physiological Mechanisms OF IL-1 Signaling Inhibition

As aberrant inflammation can lead to a myriad of pathological effects, regulation of these signaling systems is crucial for a functioning immune system. Dysregulated IL-1 signaling can mediate numerous auto-inflammatory diseases (50). There are several mechanisms to regulate IL-1 family signaling effectively (Figure 1). Within the IL-1 family of cytokines, there exist antagonist cytokines (IL-1Ra & IL-36Ra), immunosuppressive cytokines (IL-37 & IL-38), decoy receptors (sST2 and IL-1RII) and the IL-18 binding protein (IL-18BP).

### Antagonists and Immunosuppressive Cytokines

Antagonist cytokines function by binding the primary receptor and prohibiting the recruitment of the secondary receptor. This inhibits signaling by occupying the binding pocket of the primary receptor, thus not allowing an agonist cytokine the opportunity to bind. Antagonist cytokines within the IL-1 family include the IL-1 receptor antagonist (IL-1Ra), whose cognate receptor is IL-1RI, and the IL-36 receptor antagonist (IL36Ra), whose cognate receptor is IL-36R.

### IL-1Ra

**Figure 8:**
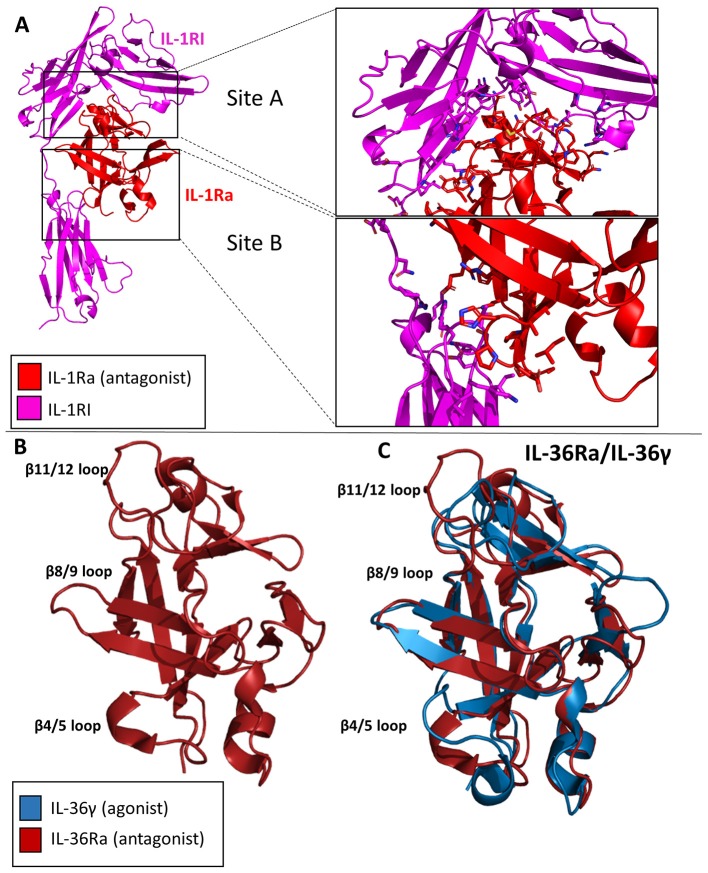
Crystal structures of IL-1 family antagonists. **(A)** IL-1Ra bound to IL-1RI (pdb: 1IRA). Site A and Site B are enlarged to display the larger interface for Site A by IL-1Ra than for Site B, contrary to the binding mechanism of IL-1β. **(B)** Crystal structure of Il-36Ra. **(C)** Crystal structure of IL36Ra aligned to crystal structure of Il-36γ.

The IL-1Ra structure was solved to a resolution of 2.1 Å by X-ray crystallography in 1994 (51). IL-1Ra is a 17 kDa polypeptide composed of 12 β-strands and two very short 3–10 helices, similar in architecture to IL-1α and IL-1β (Figure 8A) (4, 52).

IL-1Ra is an antagonist cytokine; it can occupy the binding pocket of IL-1RI without eliciting any downstream signaling (53, 54). IL-1Ra is capable of binding IL-1RI with equal affinity to IL-1RI as IL-1β, thus competing with IL-1 signaling. IL-1Ra preferentially binds IL-1RI over IL-1RII, thus not binding the decoy receptor in what would be a non-productive mechanism of inhibition. As previously stated, IL-1β binds IL-1RI at two distinct sites, sites A and B. IL-1Ra, however, binds predominantly site A, as determined by extensive mutagenesis (55). Differences also exist between Il-1β and IL-1Ra s in the β4/5 loops of the cytokines, key mediators of interaction with IL-1RAcP. The total RMSD of IL-1Ra and IL-1β is only 0.90 Å, however.

### IL-36Ra

Like IL-1Ra, IL-36Ra binds to its primary receptor and does not allow a functioning signaling complex to be formed (35). To date, many studies have shown that IL-36Ra is able to inhibit IL-36γ stimulated NF-κB signaling (33, 38, 56). The structure of murine IL-36Ra was first published in 2003 to a resolution of 1.6 Å (57). Like other cytokines within this family, IL-36Ra is composed of 12 β-strands that fold into a β-trefoil conformation (Figure 8B). The largest differences between IL-36γ and IL-36Ra were the β4/5 and β 11/12 loops (Figure 8C). To investigate the structural determinants of IL-36Ra, the loops from IL-36Ra were swapped into IL-36γ. In the case of β11/12, the inclusion of this loop from IL-36Ra into IL-36γ led to a 14-fold decrease in binding affinity and a 1,000-fold decrease in activity during *in vitro* assays (38). Swapping the β4/5 loops of IL-36Ra into IL-36γ led to a 10-fold decrease in signaling and only a slight decrease in binding affinity, highlighting that β4/5 loops may not make as crucial of interactions with the IL-1RAcP as does the β11/12 loops of IL-36γ.

### IL-38

**Figure 9:**
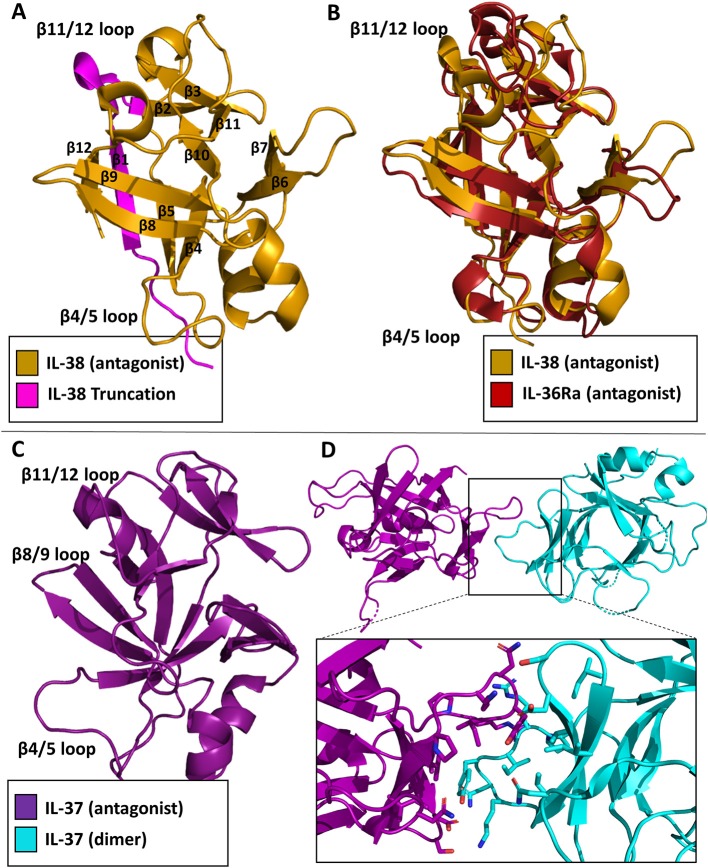
Inhibitory cytokine crystal structures. **(A)** IL-38 crystal structure (pdb: 5BOW) The truncation variant missing the first 19 amino acids can be visualized by the exclusion of the magenta portion of the protein. **(B)** Overlay of the IL-38 crystal structure with IL-36Ra (pdb: 1md6). **(C)** IL-37 crystal structure (5HN1). **(D)** Homo-dimer crystal structure of IL-37 with a focus on the dimer interface.

While the functional role of IL-38 continues to be elucidated, the structure has been deposited in the Protein Data Bank (PDB 5BOW). IL-38 was first cloned and added as a member for the IL-1 superfamily in 2001 (58). It is associated with the clinical manifestations of systemic lupus erythematosus (59). It is predominately expressed in the skin and in proliferating B cells. This cytokine lacks a signal peptide, is 152 AA in length, and does not contain any caspase-1 cleavage sites. Functionally, IL-38 inhibits *Candida albicans*-induced Th17 responses in PMBCs (60). It has been suggested that IL-38 acts through IL-36R in a fashion similar to IL-36Ra, although low affinity binding to IL-1RI has also been reported (58, 60). Moreover, a truncation variant lacking the first 19 amino acids was reported to act through IL-1RAPL1 (61). This N-terminal truncation would lead to a complete loss of beta strand 1 and part of beta strand 2 (Figure 9A), which likely leads to an unstable, misfolded and/or dysfunctional protein. How exactly IL-38 exerts its anti-inflammatory actions remains to be clarified, although it has been suggested IL-38 could recruit one of the inhibitory co-receptors of the IL-1 family, namely SIGGIR, TIGIRR1, and/or TIGIRR2 (62).

As with the other cytokines within the IL-1 family, IL-38 shares certain canonical traits. It has 12 β-strands connected by 11 loops, organized into a β-trefoil configuration (Figure 9A). Based on sequence comparison, IL-38 is related to two other well-known antagonists, IL-1Ra and IL-36Ra, with sequence identities of 39 and 43%, respectively (60). Comparison of their X-ray structures show that IL-38 has an RMSD of 1.23 Å to IL-1Ra and 0.96 Å to IL-36Ra. Superposition of IL-38 and IL-36Ra reveal that loop of β4/5, important for antagonism of IL-1Ra and IL-36Ra, is nearly identical to that of IL-36Ra (Figure 9B), further indication of IL-38's immunomodulatory role.

### IL-37

IL-37 was first identified within the IL-1 gene cluster in 2001 (63). There are five isoforms of IL-37, IL-37a-e; IL-37b is the largest with five of the six exons from the locus (64). IL-37 has proven to be a potent anti-inflammatory cytokine (65). Like IL-1β, it requires caspase 1 cleavage for activation. Transgenic expression of IL-37 abrogates inflammation in the presence of endotoxin in mice that naturally lack IL-37 (66). Upon stimulation in RAW cells, IL-37 was highly potent in reducing TNF, MIP-2, and IL-1α levels (64). Functionally, IL-37 works in two distinct ways: by trafficking to the nucleus and blocking Smad3 activation after LPS stimulation, or by interacting with the receptor chain IL-18Rα and the single Ig domain receptor SIGIRR (67). This was highlighted by the silencing of IL-18Rα and, ultimately, the reduction of the anti-inflammatory properties of IL-37 (65). Additionally, over-production of IL-37 protected mice from endo-toxemia, colitis, obesity and metabolic syndrome, spinal cord injury, and myocardialischemia (68).

In 2017, the structure of the cytokine domain of IL-37b, the best characterized isoform, was determined to 2.25 Å (Figure 9C) (67). IL-37 crystallized as a homodimer (Figure 9D). This head-to-head dimer architecture is unique to the IL-1 family cytokine structures discussed, although IL-37 still retains its 12 β-strands and 3 helices to form the prototypical β-trefoil seen in IL-1 family cytokines. While IL-37 shares a common receptor IL-18Rα with IL-18, it is only 19% identical in sequence to IL-18. The RMSD of the Cα atoms between these two cytokines, however, is 1.64 Å (67).

### Decoy Receptors

Three decoy receptors exist in the IL-1 family: IL-1RII, sST2, and IL-18BP. While these decoy receptors all mimic the primary receptor, the mechanisms by which they inhibit signaling differ.

### IL-1RII

IL-1α and IL-1β signaling may be inhibited in two ways, by the antagonist cytokine IL-1Ra (described above) and by the decoy receptor IL-1RII. IL-1RII is similar to IL-1RI, as it is composed extracellularly of three Ig-like domains and attached to the plasma membrane by a single transmembrane α-helix. IL-RII differs, however, in that it lacks an intracellular TIR domain (Figure 1D) (69). As IL-1RII can bind IL-1 agonists, it subsequently may recruit the IL-1RAcP for the creation of the IL-1 ternary complex. As both the cytoplasmic TIRs of the primary and secondary receptor are necessary for the initiation of a signaling cascade, no signaling can occur (70).

### sST2

sST2, like its membrane bound homolog ST2, is composed of three Ig-like domains. It differs, however, as it is not attached to the plasma membrane and, as the name implies, is soluble. sST2 functions by sequestering free IL-33, thus not allowing it to bind cell-surface expressed ST2 and IL-1RAcP (Figure 1F). sST2 levels have been correlated with a number of disease states associated with a Th2 response, including systemic lupus erythematosus, asthma, idiopathic pulmonary fibrosis, and sepsis (71–74).

### IL-18BP

The IL-18 binding protein (IL-18BP) is a naturally occurring negative regulator of IL-18 signaling that sequesters free IL-18 and inhibits its binding to the 18Rα (Figure 1G). While IL-18BP is usually expressed in 20-fold higher amounts than IL-18, under certain inflammatory conditions, IL-18 may be in excess (75) IL-18BP was discovered when 500 liters of human urine was concentrated and subsequently passaged over an IL-18-agarose column (76). IL-18BP was ultimately shown to abrogate the ability of IL-18 to induce a Th1 response in mice treated with LPS (76). While a naturally produced negative-feedback mechanism within humans, IL-18BP has also been acquired by a multitude of poxviruses, including molluscum contagiosum virus (MCV) and orthopoxviruses (77).

**Figure 10:**
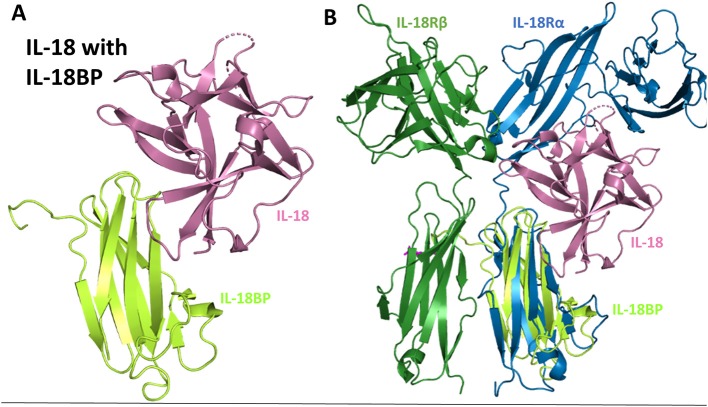
IL-18 sequestration by IL-18BP. **(A)** Crystal structure of IL-18 bound to IL-18BP (pdb 3F62). **(B)** Overlay of IL-18/IL-18BP crystal structure with ternary IL-18/IL-18Rα/IL-18Rβ to highlight IL-18BP resembling the D3 domain of IL-18Rα.

The structure of IL-18BP has been determined to 2 Å resolution (77). IL-18BP has an Ig domain with two four-stranded β-sheets and two disulfides that hold the beta-sandwich together (Figure 10A). When this complex structure is overlaid with the binary IL-18 receptor, it is evident that IL-18BP clearly adopts a binding mode resembling the D3 domain of IL-18Rα (Figure 10B). In its binding, it lies on top of β-barrel of IL-18 and adds its hydrophobic residues to the binding pocket (77). This complex has a buried surface of 1,930 Å^2^, as opposed to the 600 Å^2^ shared by IL-18 and site B of IL-18Rα (77). This would allow both the human expressing the IL-18BP or a virus that has hijacked this mechanism to downregulate IL-18 induced IFN-γ production effectively.

## Therapeutics

Dysregulation of IL-1 family signaling can result in myriad pathologies. As such, stemming the inflammatory signals inherent to agonists within the IL-1 superfamily of cytokines is an attractive therapeutic target. To date, numerous avenues of inhibiting IL-1 family signaling have been explored.

### Receptor Antagonists

**Figure 11:**
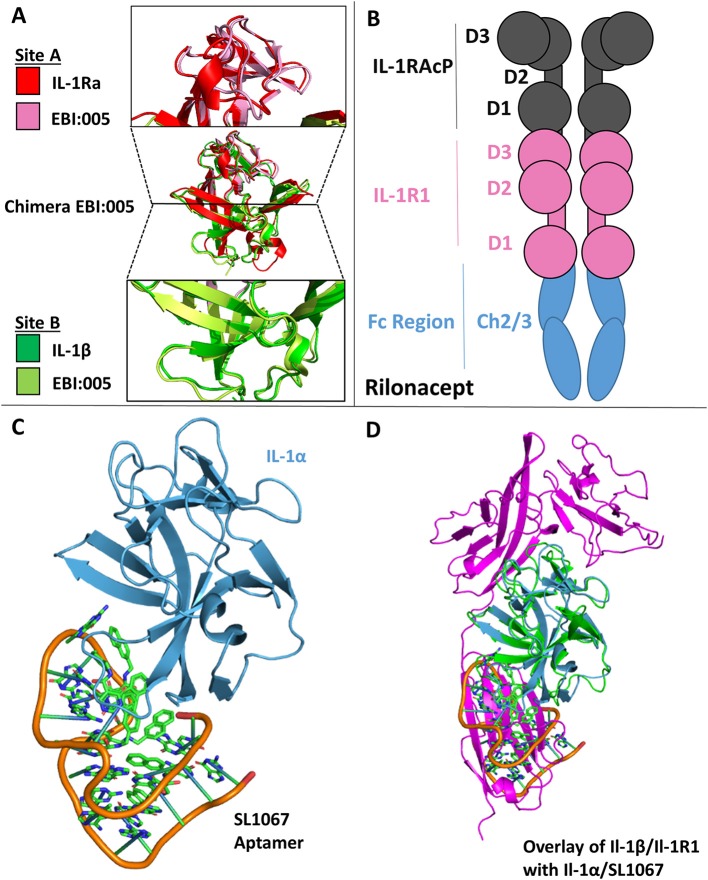
Therapeutics. **(A)** Crystal structure of Chimera EBI-005. **(A)** Top: overlay of IL-1Ra (red) and EBI:005 chimera (pink/portion of binding Site A); Middle: overlay of IL-1Ra/EBI:005/IL-1β; Bottom: Overlay of IL-1β (green) and EBI:005 chimera (lime/portion of binding Site B). **(B)** Rilonacept cartoon with respective components labeled. **(C)** Crystal structure of IL-1α bound of SL1067 aptamer (pdb: 5 uc6). **(D)** Overlay of IL-1α/SL1067 and structure IL-1β/IL-1RI (pdb: 1itb).

One of the earliest therapeutics developed against IL-1 signaling is Anakinra, the recombinant version of IL-1Ra and the first to get FDA approval (2001) (15, 78). In an effort to enhance the therapeutic potency of IL-1Ra by leveraging the insight gained from the structures of the IL-1RI/1Ra and IL-1RI/IL-1β complexes, a chimera of IL-1β/IL-1Ra was designed that bound IL-1RI with an 85-fold increase in affinity (SPR) and ~100-fold increase in potency *in vivo* (13). While the receptor antagonist has changes in the β4/5 and β11/12 loops of the cytokine, it preferentially binds site A while not binding site B, a contributing factor to its inability to make a functioning signaling complex. In contrast, IL-1β engages both sites A and B, but compared to IL-1Ra has a lower affinity for site A. Through rational protein engineering derived from structural knowledge gained from these binary complexes, the authors combined site A of IL-1Ra with site B of IL-1β to create a novel antagonist, EBI-005 (13, 15). The disassociation constant to IL-1RI of this chimera was 6.3 ×10^−6^ s^−1^ compared to 3.0 ×10^−5^ s^−1^ for IL-1Ra, leading to a theoretical half-life of 31 and 6.4 h, respectively (13). This potency was recapitulated *in vivo*, resulting in a 100-fold increase in potency of EBI-005 as compared to IL-1Ra (13). While these were not consecutive amino acid substitutions in the primary structure, these residues lie next to each other three dimensionally upon the axis of the β-trefoil (Figure 11A).

### Decoy Receptors

As described above, there are three naturally occurring decoy receptors in the IL-1 superfamily: IL-1RII, sST2, and IL-18BP (3). In addition, it has been found that a soluble version of IL-1RI may exist, as evidenced by Raji cells stimulated by dexamethasone increased surface expression of IL-1RI and, ultimately, release of a soluble version of IL-1RI(79). While a soluble version of IL-1RI had been tested in clinical trials for graft vs. host disease (GVHD), human immunodeficiency virus (HIV) and rheumatoid arthritis (RA), however, those studies were halted as no therapeutic benefit was observed. It was hypothesized that soluble IL-1RI preferentially bound to IL-1Ra and thus negated any gains that might have been seen by its anti-inflammatory properties (15).

A unique solution to the soluble receptor problem was the idea of cytokine traps, most notably the fusion peptide Rilonacept (trade name Arcalyst, Regeneron Pharmaceuticals) (80, 81). Rilonacept is the inline fusion protein of IL-1RAcP, IL-1RI, and IgG-Fc creating homodimers containing two IL-1RAcP and IL-1RI molecules (Figure 11B). In addition to its ability to neutralize IL-1 signaling by acting as a decoy receptor, cytokine traps configured in this way have the added benefit of increased therapeutic half-life due to its fusion to an IgG Fc region (81).

### DNA Aptamers

A more recent approach to countering inflammatory diseases resulting from IL-1 signaling has been the use of DNA aptamers (from the Latin “aptus,” fit; and the Greek “meros,” part), which are oligonucleotide fragments that can bind protein targets. The DNA aptamer SL1067 binds IL-1α and disrupts its ability to bind to its cognate receptor IL-1RI (Figure 11C) (82). The SL1067/IL-1α interface is composed predominately of hydrophobic moieties on both the cytokine and the aptamer, with the addition of π-interactions between amino acids and nucleotides. SL1067 binds on the surface of IL-1α that interacts with D3 of the IL-1RI. While a crystal structure of the IL-1α/IL-1RI binary complex has not been published to date, the RMSD between IL-1α and IL-1β is 1.56 Å and thus can be superimposed to the IL-1β/IL-1RI binary complex for visualization (Figure 11D), indicating its mechanism of action (82).

### Peptides

While naturally occurring receptor antagonists work well at abrogating IL-1 signaling, an early goal of the field was to discover lower molecular weight antagonist peptides that could be delivered orally to patients. As early as 1996, numerous peptides that inhibited IL-1RI signaling had been discovered by phage display (83). One such peptide, AF10847, was crystalized with IL-1RI to determine its mechanism of antagonism (84), showing that this peptide bound site A of IL-1RI and induced a conformational change in the receptor that renders it incapable of cytokine binding. Indeed, binding site B swung ~170 degrees away from the orientation of IL-1β binding, thus not allowing agonist binding and at the same time demonstrating the flexibility of the D3 domain in respect to D1/2 (84).

### Antibodies

An early solution to aberrant IL-1 family signaling by agonist cytokines was the development of neutralizing antibodies to these potent mediators of inflammation. AMG108 is currently an antibody licensed to AstraZeneca and targets IL-1RI. It has been shown that this antibody can block IL-1 mediated signaling and is efficacious in the treatment of osteoarthritis (85). More commonly, monoclonal antibodies have been made against agonist cytokines, such as IL-1α and IL-1β, to stem aberrant inflammatory signaling. While there are currently multiple disease states that anti-IL-1β antibodies are being used against, Canakinumab was approved by the FDA for the treatment of cryopyrin-associated periodic syndromes (CAPS) in 2009 (15). This methodology has also been applied to IL-1α, as with the case of MABp1, a mAb targeting refractory cancers (86). As inflammation is responsible for a number of pleiotropic disease states, antibodies against IL-1 family signaling is an attractive target. As there exists numerous pharmaceutical antibodies at various stages of development, the entirety of the known repertoire will not be addressed in this review.

## Future Perspectives

There remains much to be learned within the IL-1 family. No binary or ternary structures involving IL-36 cytokines have been determined at any resolution. As such, the interaction of IL-36 with its primary receptor and recruitment of IL-1RAcP remains poorly understood. As was learned with IL-1 and IL-33, an altogether unique mode of Il-1RAcP interaction may yet exist for IL-36 agonist cytokines. In addition, the precise mechanisms of IL-37 and IL-38 remain to be determined. IL-37 supposedly has a novel mechanism of negative regulation of itself, dimerizing above a certain concentration threshold (67). This could prove particularly difficult for solving a ternary complex of IL-37 by crystallography as reaching metastable concentrations of the respective components might prevent ternary complex formation. While preliminary data has been published concerning its function and primary receptor, high resolution data addressing its dependence on IL-18Rα and SIGIRR are yet missing.

There are two more orphan receptors in the IL-1 family, IL-1 receptor accessory protein like (IL-1RAPL) 1 and 2. Based on structural similarity, they are grouped with IL-1RAcP (87). No immunological function has yet been attributed to them. Instead, they were shown to play an important role in the neuronal system in trans-synaptic signaling (88). The ectodomain of IL-1RAPL1 binds the ectodomain of protein tyrosin phosphatase receptor δ (PTPRδ). Additionally, IL-1RAcP was shown to facilitate trans-synaptic signaling in a similar way (89). Crystal structures of both IL-1 family receptors with PTPRδ revealed that mainly the D1 domain of both IL-1AcP and IL-1RL1 was engaged by PTPRδ (87). Why IL-1RAcP functions in both the immune and nervous systems remains unclear. Notably, there is a unique isoform of IL-1RAcP only found in the nervous system (90).

Beyond the extracellular domains of IL-1 family receptors, the particulars of the intracellular signaling cascade remain somewhat of a mystery in regards to their actual mechanistic interactions with other TIRs, such as MyD88. Cytoplasmically, receptors of the IL-1 family are attached to TIR domains through a single trans-membrane helix. Analogous to the Toll-Like-Receptor (TLR) TIR domains, these mediators bind several cytoplasmic molecules to initiate intracellular signaling. To date, the structure of only a single TIR domain from the IL-1 family has been solved. The first IL-1 family TIR domain structure determined was a homo-dimer of IL-1 receptor accessory protein like 1 (IL-1RAPL1) in 2004 (91). As there are significant differences in both sequence identity and structural similarity to other known TIR domains, namely the TLR TIR domains, it suggests that TIR structural diversity allows for the diverse signal transduction that can occur. In coming years, IL-1 family TIR domain structural studies could provide a fruitful avenue of research for the field. As was learned through the ectodomains of these receptors, the differences inherent to the TIRs mediate their action and could prove attractive therapeutic targets.

Structural biology has added a wealth of information to the molecular mechanisms of IL-1 family signaling. To date, three high-resolution ternary complexes have been determined by X-ray crystallography: IL-1β/IL-1R1/IL-1RAcP, IL-33/ST2/IL-1RAcP, and Il-18/IL-18Rα/IL-18Rβ. Through these structures, the distinctive structural and functional properties of each respective ternary complex have been elucidated. As such, new avenues of antibody therapy are now clear. As previously stated, both IL-1β and IL-33 binary complexes recruit the IL-1RAcP secondary receptor differently. By targeting different solvent accessible features of IL-1RAcP, and thus different interface residues, selective inhibition of IL-1 family signaling cascades might be achieved. This could be especially useful for IL-33 as no natural antagonist cytokine exists for the primary receptor.

The field's structural biology knowledge can be leveraged in other ways as well. In addition to antagonist cytokines, it is possible to use low molecular weight peptides that bind the primary receptor and act as antagonists (84). The flexibility of the D3 domain with respect to D1/2 has been shown experimentally (40) as well as theoretically (92, 93). By using the inherent flexibility of the primary receptor by holding the primary receptor in a non-amenable conformation for cytokine binding, IL-1 family signaling can be ablated.

## Conclusion

IL-1 family signaling is an instrumental component of an inflammatory response, ultimately helping to orchestrate both innate and adaptive immunity to fight a myriad of pathogens. Conversely, aberrant signaling within these systems can lead to a host of auto-inflammatory disease states. By employing methods in structural biology, a wealth of information has been gained concerning how these receptor complexes function and the particulars of each subfamily system. Through the knowledge that has and will continue to be learned through structural biology, it will be possible to fully understand these member systems and, feasibly, harness it for our therapeutic benefit.

## Author Contributions

JF wrote the manuscript and designed the figures. SG and ES edited the manuscript and the figures.

### Conflict of Interest Statement

The authors declare that the research was conducted in the absence of any commercial or financial relationships that could be construed as a potential conflict of interest.
